# Comparative effectiveness of two antimicrobial regimens for the treatment of pan-drug-resistant *Acinetobacter baumannii* infections: results from the DESPAIR study

**DOI:** 10.1128/aac.01154-25

**Published:** 2026-01-15

**Authors:** Konstantinos Pontikis, Ilias Karaiskos, Aikaterini Sakagianni, Eleni Mouloudi, Evdoxia Tsigou, Aikaterini Gkoufa, Sofia Michelidou, Kristina Mouratidou, Alexandra Gavala, Christina Routsi, Stamatis Karakonstantis, Stavroula Michalea, Sevasti Ampelioti, Theodora Katsarou, Athanasios Papathanasiou, Maria Pirounaki, Charalambos Anastogiannis, Antonia Koutsoukou, Helen Giamarellou, George L. Daikos, on behalf of the DESPAIR Study Group

**Affiliations:** 1 Chest Diseases Hospital ‘I Sotiria’, Intensive Care Unit 1st Department of Pulmonology, National and Kapodistrian University of Athens 68993, Athens, Greece; 2 1st Department of Internal Medicine - Infectious Diseases, Hygeia General Hospital 69047, Athens, Greece; 3 Intensive Care Unit, Sismanogleio General Hospital of Attica 72864 , Athens, Greece; 4 Intensive Care Unit, Hippokration General Hospital of Thessaloniki 69200 , Thessaloniki, Greece; 5 Intensive Care Unit, General and Oncologic Hospital of Kifisia ‘Oi Agioi Anargyroi’ 69069 , Athens, Greece; 6 1st Department of Medicine, School of Medicine, Laiko General Hospital, National and Kapodistrian University of Athens 68989, Athens, Greece; 7 Evangelismos General Hospital 1st Department of Intensive Care Medicine, National and Kapodistrian University of Athens 68993, Athens, Greece; 8 Internal Medicine Department, University Hospital of Heraklion, Heraklion, Greece; 9 Intensive Care Unit, Korgialenio-Mpenakio General Hospital 168201 , Athens, Greece; 10 Intensive Care Unit, Chest Diseases Hospital ‘I Sotiria’ 221171 , Athens, Greece; 11 Intensive Care Unit, University Hospital of Ioannina 69157 , Ioannina, Greece; 12 2nd Department of Internal Medicine, School of Medicine, Hippokration General Hospital, National and Kapodistrian University of Athens 68989, Athens, Greece; 13 Intensive Care Unit, Aghios Andreas Hospital 567848 , Patras, Greece; 14 2nd Department of Internal Medicine, Mitera General Hospital 69036 , Athens, Greece; University of Fribourg, Fribourg, Switzerland

**Keywords:** pan-drug-resistant *Acinetobacter baumannii*, triple antibiotic therapy, colistin, meropenem, tigecycline, antimicrobial resistance

## Abstract

Pan-drug-resistant *Acinetobacter baumannii* (PDR-AB) causes severe infections and constitutes a threat in several geographic regions. Little is known about the differential effectiveness of last-resort regimens, frequently used in this setting. We compared the effectiveness of two literature-proposed regimens against PDR-AB infections consisting of colistin, ampicillin-sulbactam, and either meropenem (regimen A) or tigecycline (regimen B). This is a retrospective analysis of prospectively collected data from 12 centers on adult patients with hospital-acquired pneumonia (HAP) or bloodstream infection (BSI), who had received definitive treatment with either regimen A or regimen B. The primary outcome was clinical failure, defined as any of the following occurring by day 14 from infection onset: death, initiation of salvage treatment, treatment withdrawal due to toxicity, persistent bacteremia for BSI patients and failure to improve oxygenation for HAP patients before and after propensity matching. Eighty-three patients were included in the primary analysis; 60 had received regimen A and 23 regimen B. Regimen B was significantly associated with clinical failure before and after propensity matching (odds ratios [OR]: 3.11; 95% confidence interval [CI]: 1.10–8.84 vs OR: 3.83; 95% CI: 1.26–11.63), respectively. Salvage therapy and treatment discontinuation due to toxicity were more frequent in patients treated with regimen B. In multivariable analysis, regimen B was independently associated with 28-day mortality before (hazard ratio [HR]: 2.53; 95% CI: 1.08–5.94) but not after propensity matching (HR: 2.64; 95% CI: [0.99–7.02]). Treatment with colistin, ampicillin-sulbactam, and meropenem against severe PDR-AB infections was associated with favorable outcomes compared to colistin, ampicillin-sulbactam, and tigecycline.

## INTRODUCTION

Treatment of carbapenem-resistant *Acinetobacter baumannii* (CRAB) infections is acknowledged by the World Health Organization as a field of vital importance. Along with carbapenem-resistant or third-generation cephalosporin-resistant *Enterobacterales* and rifampicin-resistant *Mycobacterium tuberculosis,* CRAB forms the “critical priority group” due to its “virulence, resistance, and limited treatment options, leading to severe nosocomial infections, especially among intensive care patients, and alarmingly high mortality rates” (1).

The polymyxins have been the cornerstone of antimicrobial treatment of CRAB infections (2–4) for more than 20 years (5). However, resistance has emerged and poses significant threats to successful therapy (6). Initial reports of colistin resistance date back to the late 1990s (7). Since then, colistin non-susceptibility has disproportionately increased in certain regions and nosocomial environments, especially those associated with critical care. Recent evidence suggests that several countries have been inflicted (8). In Greece, according to a recent report by the Greek Antimicrobial Resistance Surveillance System, resistance to colistin among *A. baumannii* blood isolates exceeded 40% (9). Frequently enough, polymyxin resistance is synonymous with pan-drug resistance (10), especially since there are no established breakpoints for sulbactam and tigecycline.

Optimal treatment of pan-drug-resistant *A. baumannii* (PDR-AB) infections has not been defined. Despite evidence that combination treatment is not beneficial against CRAB infections (11, 12), most reports on PDR-AB infection treatment describe double or triple combinations of inactive *in vitro* agents. Among these regimens, two have taken precedence, at least anecdotally, in Greece. The first is based on a report by Qureshi et al. (13) and contains colistin, a carbapenem, and ampicillin-sulbactam. In this study, this regimen was associated with the lowest mortality rate in a cohort of 20 patients with severe colistin-resistant *A. baumannii* infections (13). The second is based on Assimakopoulos et al. (14), contains colistin, tigecycline, and ampicillin-sulbactam and was associated with a favorable clinical outcome in 9/10 patients with colistin-resistant *A. baumannii* ventilator-associated pneumonia (14).

Under these pressing circumstances, the Hellenic Society of Antimicrobial Chemotherapy (HSCH) opted for organizing an observational study on CRAB infections, with a special focus on the PDR phenotype (dealing with severe problematic drug-resistant *Acinetobacter baumannii* infections regionally [DESPAIR]).

## RESULTS

**Fig 1:**
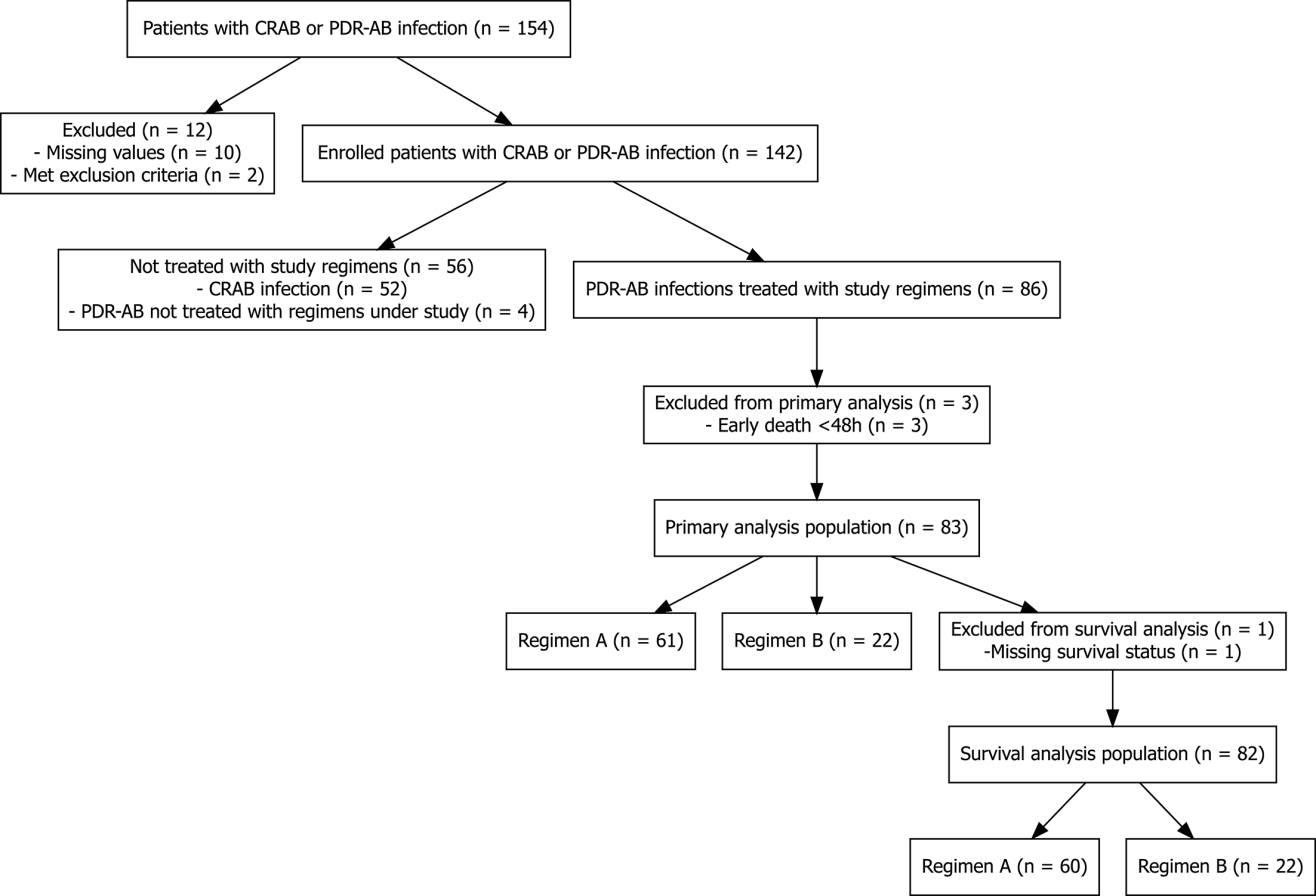
Study flow chart.

The study flow chart is presented in Fig. 1. Between February 2022 and September 2024, 154 patients with CRAB infections were enrolled. Among them, 86 patients with PDR-AB infections from 12 sites in 11 hospitals were suitable for the current analysis. Each site contributed between 2 and 20 cases. Sixty-one patients received regimen A, while 25 patients received regimen B. The two patient groups were comparable in terms of demographic characteristics, medical history, and features of the index infection (Table 1; Table S1). However, a notable difference was observed in the initial clinical status of patients at infection onset; a significantly higher proportion of patients in group A were admitted to the intensive care unit (ICU) at the time of enrollment (93% vs 68% in group B). Consistent with this disparity, group A also had a greater proportion of patients requiring mechanical ventilation and a higher frequency of ventilator-associated pneumonia (VAP) among pneumonia cases (Table 1). Specifically, 66 patients suffered from pneumonia with the percentage of VAP being 85% and 53% in groups A and B, respectively.

**TABLE 1:** Demographics, history, index infection, and therapy characteristics among treatment groups^*a*^

Characteristic		Group A (*n* = 61)	Group B (*n* = 25)	Total	*P* value
Age, median (IQR)		72.0 (58.5–77.5)	68.0 (59.0–77.5)	69.5 (58.8–77.3)	0.808
Female gender, n (**%**)		23 (38)	12 (48)	35 (41)	0.378
In ICU at enrollment, n (%)		**57** (**93**)	**17** (**68**)	**74** (**86**)	**0.002**
Community residence, n (**%**)		59 (97)	24 (96)	83 (97)	0.869
COVID-19 admission, n (%)		12 (20)	2 (8)	14 (16)	0.183
Immunosuppression, n (**%**)		7 (12)	1 (4)	8 (9)	0.278
Diabetes mellitus, n (%)		17 (28)	9 (26)	26 (30)	0.211
Malignancy, n (%)		11 (18)	4 (16)	15 (18)	0.474
Moderate severe CKD, n (%)		9 (15)	2 (8)	11 (13)	0.394
Congestive heart failure, n (%)		9 (15)	5 (20)	14 (16)	0.550
Coronary heart disease, n (%)		11 (18)	7 (28)	18 (21)	0.302
COPD, n (%)		18 (30)	5 (20)	23 (27)	0.366
McCabe-Jackson classification, n (**%**)					
Non-fatal disease		48 (79)	17 (68)	65 (76)	0.574
Ultimately fatal disease		10 (16)	6 (24)	16 (19)	
Rapidly fatal disease		3 (5)	2 (8)	5 (6)	
Charlson comorbidity index, median (IQR)		5.0 (3.0–6.0)	4.0 (2.3–6.8)	5.0 (3.0–6.3)	0.803
APACHE II score, mean (SD) (*N* = 85)		20.7 (7.6)	20.4 (8.4)	20.6 (7.8)	0.862
Infection type, n (%)					
BSI		14 (23)	6 (24)	20 (23)	0.917
HAP/VAP		47 (77)	19 (76)	66 (77)	
Time from ICU admission for ICU patients (days, *N* = 74), median (IQR)		8.0 (4.0–14.0)	7.0 (1.0–21.0)	8.0 (3.0–19.0)	0.742
Time from hospital admission for ward patients		8.5 (1.0–41.5)	25.5 (4.5–43.5)	18.5 (0.5–49.8)	0.683
Pneumonia type, n (%) (*N* = 66)					
VAP		**40 (85)**	**10 (53)**	**50 (76)**	**0.018**
vHAP		**3 (6)**	**5 (26)**	**8 (12)**	
HAP		**4 (9)**	**4 (21)**	**8 (12)**	
Bacteremic pneumonia, n (%) (*N* = 66)		27 (57)	12 (63)	39 (59)	0.669
Sepsis, n (%)		28 (46)	9 (36)	37 (43)	0.124
Septic shock, n (%)		25 (41)	8 (32)	33 (38)	
Vasopressor use, n (%)		39 (64)	12 (48)	51 (59)	0.172
Invasive MV, n (%)		**49 (80)**	**14 (56)**	**63 (73)**	**0.021**
CRRT, n (%)		10 (16)	2 (8)	12 (14)	0.308
SOFA score, median (IQR)		8 (5–10)	7 (3–11)	8 (4–10)	0.272
Lactate (mEq/L), median (IQR) (*N* = 82)		1.6 (1.0–1.8)	1.0 (0.9–1.6)	1.4 (1.0–1.8)	0.163
Creatinine (mg/dL), median (IQR)		1.0 (0.6–1.5)	0.8 (0.7–1.4)	0.9 (0.7–1.4)	0.689
Albumin (g/dL), median (IQR) (*N* = 73)		2.9 (2.6–3.4)	2.8 (2.4–3.0)	2.9 (2.5–3.2)	0.257
WBC (*10^3^/μL), mean (SD)		14.6 (7.1)	13.3 (6.7)	14.3 (6.9)	0.430
Empiric treatment administered		56 (92)	20 (80)	76 (88)	0.121
Number of empiric antimicrobials per patient		2.0 (1.0–2.0)	2.0 (1.0–2.0)	2.0 (1.0–2.0)	0.355
Time from infection onset to empiric treatment per antibiotic, median (IQR)		0.0 (0.0–0.0)	0.0 (0.0–0.0)	0.0 (0.0–0.0)	0.549
Duration of empiric treatment per antibiotic (days), median (IQR)		3.0 (2.0–3.5)	3.0 (2.0–4.0)	3.0 (2.0–4.0)	0.327
Receipt of active empiric regimen, n (%) (*N* = 67)		0 (0)	0 (0)	0 (0)	N/A
Time to definitive colistin (days), median (IQR)		3.0 (2.0–3.0)	3.0 (1.6–3.5)	3.0 (3.0–3.0)	0.802
Time to definitive ampicillin-sulbactam (days), median (IQR)		3.0 (2.0–3.0)	3.0 (2.0–4.0)	3.0 (2.0–4.0)	0.450
Time to definitive meropenem (days), median (IQR)		3.0 (2.0–3.0)	N/A	3.0 (2.0–3.0)	N/A
Time to definitive tigecycline (days), median (IQR)		N/A	3.0 (2.0–4.0)	3.0 (2.0–4.0)	N/A
Colistin daily maintenance dose (million IU), median (IQR)		9.0 (6.0–10.5)	9.0 (6.0–9.0)	9.0 (6.0–10.0)	0.983
Ampicillin-sulbactam daily dose (g), median (IQR)		24.0 (18.0–27.0)	24.0 (12.0–27.0)	24.0 (18.0–27.0)	0.822
Meropenem daily dose (g), median (IQR)		6.0 (4.0–6.0)	N/A	N/A	N/A
Tigecycline daily maintenance dose (mg), median (IQR)		N/A	200 (150–200)	N/A	N/A
Colistin treatment duration (days), median (IQR)		12.0 (8.0–14.0)	11.5 (9.0–20.0)	12.0 (8.5–14.5)	0.580
Ampicillin-sulbactam treatment duration (days), median (IQR)		12.0 (8.0–14.0)	11.0 (9.0–23.3)	12.0 (8.5–14.0)	0.614
Meropenem treatment duration (days), median (IQR)		11.0 (8.0–13.0)	N/A	N/A	N/A
Tigecycline treatment duration (days), median (IQR)		N/A	11.0 (5.3–18.8)	N/A	N/A

^*a*^ Bold fonts correspond to statistically significant differences. McCabe-Jackson classification follows the guidance of the European Centre for Disease Prevention and Control (15), where non-fatal disease, ultimately fatal disease, and rapidly fatal disease have expected survival of at least 5 years, between 1 and 5 years, and less than 1 year, respectively. Group A: colistin, ampicillin-sulbactam, meropenem. Group B: colistin, ampicillin-sulbactam, tigecycline. IQR, interquartile range; ICU, intensive care unit; COVID-19, coronavirus disease-2019; CKD, chronic kidney disease; COPD, chronic obstructive pulmonary disease; APACHE II, Acute Physiology and Chronic Health Evaluation score II; SD, standard deviation; BSI, bloodstream infection; HAP, hospital-acquired pneumonia; VAP, ventilator-associated pneumonia; vHAP, ventilated HAP; MV, mechanical ventilation; CRRT, continuous renal replacement therapy; SOFA, sepsis-related organ function assessment score; WBC, white blood cells; N/A, not applicable.

All pathogens were resistant to colistin. In almost all isolates, colistin susceptibility was tested via broth microdilution (93% and 96% in groups A and B, respectively). Automated systems were employed in three cases (5%) in group A and one case (4.5%) in group B, and diffusion strips in one case (2%) in group A. In four cases, the method was unknown. Empiric antimicrobial treatment was instituted in 88% of patients, immediately after the clinical onset of infection. However, all empiric regimens were inactive *in vitro*, as expected from the resistance profile of pathogens under study (Table 1). The antimicrobial daily doses were high and concordant with HSCH guidelines (Table 1; Table S2). Treatment duration was comparable among the two groups; between 11 and 12 days at a median (Table 1).

**Fig 2:**
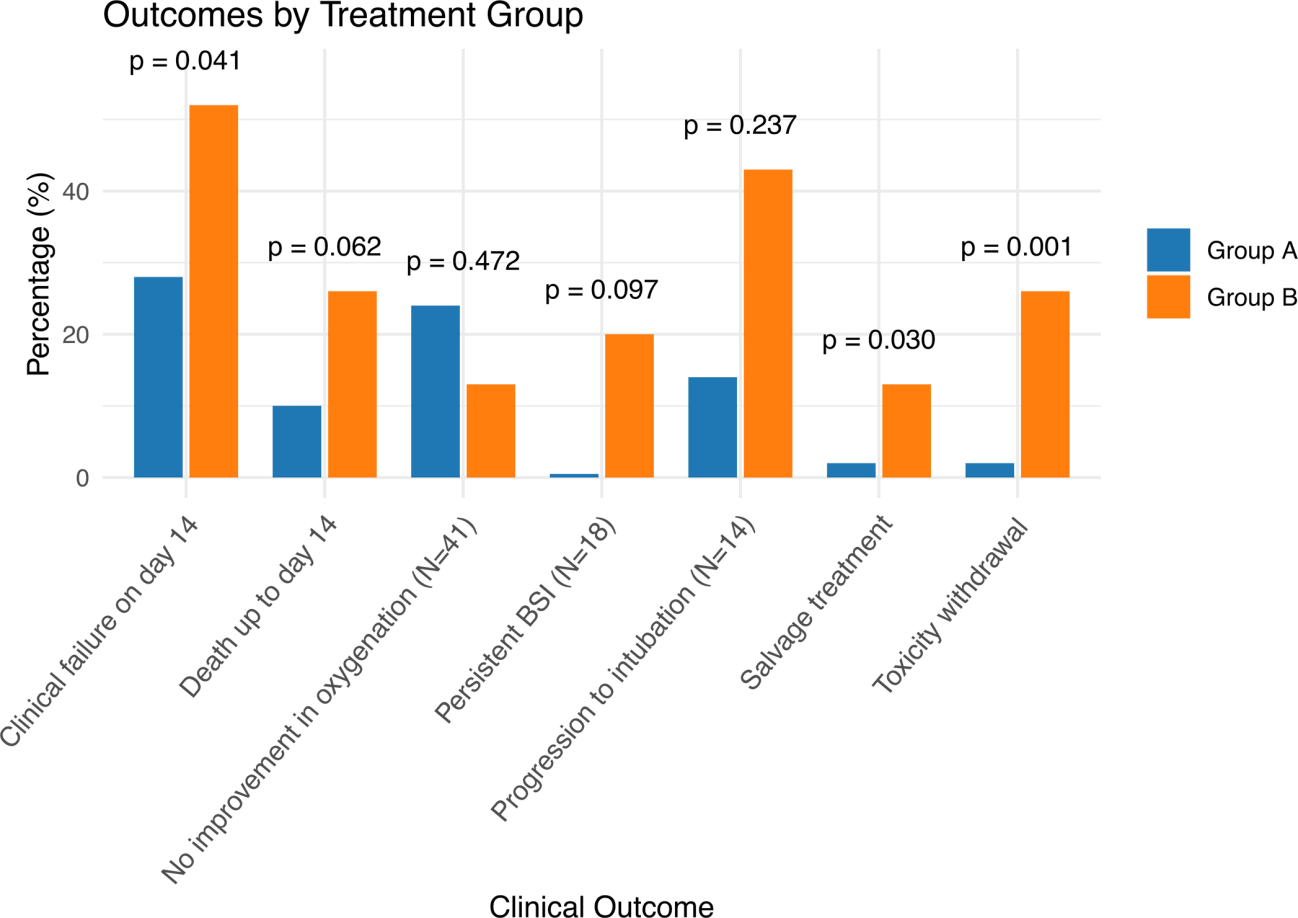
Clinical failure rates, along with elements of it, among the two treatment groups. The percentages refer to the primary analysis population (*N* = 83) unless stated otherwise. The outcome of BSI persistence was evaluated in 18 patients in whom blood cultures were taken between days 6 and 14. The criterion of absence of oxygenation improvement was evaluated in day 14 survivors who were on mechanical ventilation at study enrollment. The progression to intubation was evaluated in patients not on mechanical ventilation at study enrollment. BSI, bloodstream infection.

Three patients (one from group A and two from group B) were excluded from the primary outcome analyses due to protocol-defined early death. One additional patient (group B) was excluded from survival and resource utilization analyses due to missing data (Fig. 1). Clinical failure by day 14 was significantly more frequent in group B (12/23, 52%) than in group A (17/60, 28%) (*P*: 0.041) (Fig. 2). Results of the univariable analyses appear on Table 2. Regimen B treatment was associated with an approximately threefold increase in the odds of clinical failure (OR: 2.76 [1.02–7.44], *P* value: 0.045). This finding was also true for the multivariable analysis, where group B allocation was an independent predictor of outcome, when the effect was adjusted for established risk factors, such as the patient age, Charlson comorbidity index, and sepsis-related organ function assessment (SOFA) score, at infection onset (16) (Table 3). In the primary analysis, propensity matching via inverse probability of treatment weighting (IPTW) did not alter these results (Table 3).

**TABLE 2:** Univariable logistic regression analysis of clinical failure by day 14 from infection onset^*a*^

Characteristic		Clinical failure		Total (*N* = 83)	OR (95% CI)	*P* value
		No (*N* = 54)	Yes (*N* = 29)			
Group B, n (%)		**11 (20)**	**12 (41)**	**23 (28)**	**2.759 (1.023–7.443)**	**0.045**
Age, median (IQR)		68 (57–76)	73 (65–77)	69 (59–77)	1.019 (0.986–1.053)	0.269
Female gender, n (%)		22 (41)	13 (45)	35 (42)	1.182 (0.475–2.939)	0.719
Ward disposition (vs ICU), n (%)		7 (13)	4 (14)	11 (13)	1.074 (0.287–4.025)	0.915
COVID-19, n (%)		7 (13)	7 (24)	14 (17)	2.136 (0.667–6.839)	0.201
Immunosuppression, n (%)		4 (7)	4 (14)	8 (10)	2.000 (0.461–8.670)	0.354
McCabe-Jackson classification, n (%)						
Non-fatal disease		42 (78)	21 (72)	63 (76)	Reference	0.474
Ultimately fatal disease		8 (15)	8 (28)	16 (19)	2.000 (0.658–6.076)	0.221
Rapidly fatal disease		4 (7)	0 (0)	4 (5)	N/A	0.999
Diabetes mellitus, n (%)						
None		39 (72)	18 (62)	57 (69)	Reference	0.485
Uncomplicated		6 (11)	6 (21)	12 (15)	2.167 (0.613–7.653)	0.230
End-organ damage		9 (17)	5 (17)	14 (17)	1.204 (0.353–4.108)	0.767
Malignancy, n (%)						
None		47 (87)	22 (76)	69 (83)	Reference	0.375
Any leukemia, lymphoma, or localized solid tumor		6 (11)	5 (17)	11 (13)	1.780 (0.490–6.469)	0.381
Metastatic solid tumor		1 (2)	2 (7)	3 (4)	4.273 (0.368–49.676)	0.246
Congestive heart failure, n (%)		10 (19)	4 (14)	14 (17)	0.704 (0.200–2.480)	0.585
COPD, n (%)		12 (22)	11 (38)	23 (28)	2.139 (0.797–5.739)	0.131
Charlson comorbidity index, median (IQR)		4.0 (3.0–6.0)	5.0 (4.0–7.0)	5.0 (3.0–6.0)	1.089 (0.930–1.276)	0.291
APACHE II score, mean (SD)		**18.2 (6.6)**	**25.0 (7.1)**	**20.6 (7.5)**	**1.166 (1.072–1.267)**	**<0.001**
Infection type pneumonia (vs BSI)		**37 (69)**	**26 (90)**	**63 (76)**	**3.982 (1.057–14.995)**	**0.041**
Pneumonia type (VAP vs vHAP vs HAP), n/N (%)						
VAP		24/37 (65)	18/26 (69)	42/63 (67)	Reference	0.892
vHAP		4/37 (11)	3/26 (12)	7/63 (11)	1.000 (0.199–5.037)	1.000
HAP		9/37 (24)	5/26 (19)	14/63 (22)	0.741 (0.212–2.592)	0.639
Bacteremic pneumonia, n/N (%)		20/37 (54)	17/26 (65)	37 (59)	1.606 (0.570–4.519)	0.370
Sepsis classification, n (%)						
No sepsis		8 (15)	8 (28)	16 (19)	Reference	0.098
Sepsis		28 (52)	8 (28)	36 (43)	0.286 (0.081–1.003)	0.051
Septic shock		18 (33)	13 (45)	31 (37)	0.722 (0.215–2.427)	0.599
Vasopressors, n (%)		30 (56)	20 (69)	50 (60)	1.778 (0.686–4.608)	0.236
Invasive mechanical ventilation, n (%)		38 (70)	23 (79)	61 (74)	1.614 (0.553–4.713)	0.381
CRRT, n (%)		5 (9)	6 (21)	11 (13)	2.557 (0.707–9.251)	0.153
Lactate (mmol/L), median (IQR)		1.4 (1.0–1.8)	1.6 (1.0–2.0)	1.4 (1.0–1.8)	1.159 (0.853–1.574)	0.345
Creatinine (mg/dL), median (IQR)		**0.9 (0.4–1.0)**	**1.1 (0.7–3.2)**	**0.9 (0.6–1.4)**	**1.908 (1.199–3.037)**	**0.006**
Albumin, g/dL, mean (SD)		**3.0 (0.6)**	**2.7 (0.5)**	**2.9 (0.5)**	**0.363 (0.134–0.985)**	**0.047**
WBC, *1,000/mm^3^, median (IQR)		12.6 (9.2–16.0)	15.1 (10.0–20.5)	12.7 (9.2–17.7)	1.045 (0.979–1.115)	0.189
PaCO2, mmHg, median (IQR)		**40 (33–47)**	**45 (39–55)**	**41 (35–49)**	**1.068 (1.01–1.120)**	**0.006**
SOFA score, median (IQR)		7 (4–10)	10 (4–11)	8 (4–10)	1.079 (0.974–1.195)	0.145
Time to definitive treatment (all agents started), days, median (IQR)		3.0 (2.0–3.0)	3.0 (2.0–4.0)	3.0 (2.0–4.0)	1.036 (0.803–1.337)	0.783

^*a*^ Bold fonts denote statistical significance. Treatment group A consisted of colistin, ampicillin-sulbactam, and meropenem. Treatment group B consisted of colistin, ampicillin-sulbactam, and tigecycline. APACHE, Acute Physiology and Chronic Health Evaluation; SOFA, sepsis-related organ function assessment; CRRT, continuous renal replacement therapy; WBC, white blood cells.

**TABLE 3:** Multivariable logistic regression analysis of the primary outcome^*a*^

Variable	Odds ratio (95% CI)	*P* value
Before propensity matching		
Regimen B treatment	3.112 (1.095–8.840)	0.033
Age	1.018 (0.972–1.066)	0.460
Charlson comorbidity index	1.050 (0.844–1.306)	0.664
SOFA score	1.104 (0.989–1.232)	0.079
After propensity matching		
Regimen B treatment	3.825 (1.258–11.633)	0.018
Age	0.990 (0.942–1.040)	0.690
Charlson comorbidity index	1.120 (0.866–1.447)	0.387
SOFA score	1.048 (0.935–1.174)	0.420

^*a*^ The unadjusted model refers to the observed data. In the adjusted model, propensity matching was performed via inverse probability of treatment weighting, where the probability of receiving regimen B was modeled as a function of patient disposition, infection type, and SOFA score at infection onset. Robust standard errors (Huber-White) were implemented for model stability.

The association of treatment regimen with clinical failure withstood three sensitivity analyses. In the first, the effect of regimen B treatment was explored in a model where variable selection was based on statistical criteria and included Acute Physiology and Chronic Health Evaluation (APACHE) II score on admission, serum creatinine, and partial pressure of carbon dioxide in arterial blood on infection onset. In this analysis, the patient group was still a significant predictor of clinical failure; however, with wider confidence intervals and less model stability (Table S3). The second, where the probability of receiving regimen B was introduced as a covariate in the model containing patient group, age, infection onset SOFA score, and Charlson Comorbidity Index, also upheld the treatment effect (Table S4). Last, excluding toxicity-related withdrawal from the definition of clinical failure had no effect on the association between regimen B and outcome, both before and after propensity matching (Table S5).

The secondary outcomes are presented in Table 4 and Fig. 2; Fig. S1 and S2 and were generally in line with the primary outcome. Among elements of clinical failure, regimen B treatment was associated with more prescriptions of salvage treatment (3/23, 13% vs 1/60, 1.7%, *P*: 0.030), a greater number of drug withdrawals due to perceived toxicity (6/23, 26% vs 1/60, 1.7%, *P*: 0.001) and a trend toward more deaths up to day 14 (6/23, 26% vs 6/60, 10%, *P*: 0.062). One out of five group B patients with bloodstream infection (BSI) had microbiological persistence beyond day 5, while no occurrences of persistent infection were noted in group A (Fig. 2).

**TABLE 4:** Secondary outcomes^*a*^

Outcome	Total (*N* = 83)	Group A (*N* = 60)	Group B (*N* = 23)	*P* value
Microbiological eradication in the microbiologically evaluable population, n/N (%)^*b*^	61/79 (77)	43/57 (75)	18/22 (82)	0.545
BSI, n/N (%)^*c*^	18/19 (95)	13/13 (100)	5 /6 (83)	0.130
VAP/HAP/vHAP, n/N (%)^*d*^	43/60 (72)	30/44 (68)	13/16 (81)	0.321
Recurrent infection, n (%)	6 (7)	3 (5)	3 (13)	0.205
Superinfection, n (%)	11 (13)	7 (12)	4 (17)	0.491
Day 28 mortality, n/N (%)^*e*^	24/82 (29)	15/60 (25)	9/22 (41)	0.161
	**Total (*N* = 71)**	**Group A (*N* = 56)**	**Group B (*N* = 15)**	***P* value**
Intervention-free days in the ICU population				
ICU-free days, median (IQR)	0.0 (−1.0–9.0)	0.0 (−0.5–9.0)	0.0 (−1.0–13.0)	0.375
Ventilator-free days, median (IQR)	7.0 (−1.0–22)	9.5 (−0.5–22.0)	0.0 (−1.0–23.0)	0.247
Vasopressor-free days, median (IQR)	16.0 (−1.0–25.0)	8.5 (−0.5–25.5)	14.0 (−1.0–25.0)	0.261
CRRT-free days, median (IQR)	28.0 (−1.0–28.0)	28.0 (1.5–28.0)	18.0 (−1.0–28.0)	0.017
Organ support-free days, median (IQR)	2.0 (−1.0–19.0)	7.5 (−0.5–20.5)	0.0 (−1.0–17.0)	0.170

^*a*^ Microbiological outcomes and vital status at day 28 correspond to the total outcome analysis population, while resource utilization indices correspond to the ICU population. Denominators appear in header rows and are used in comparisons unless stated otherwise. BSI, bloodstream infection; VAP, ventilator-associated pneumonia; HAP, hospital-acquired pneumonia; vHAP, ventilated HAP; ICU, intensive care unit; CRRT, continuous renal replacement therapy.

^*b*^ Microbiological outcome was unknown in four patients, three in group A and one in group B.

^*c*^ Microbiological outcome was unknown in one BSI patient.

^*d*^ Microbiological outcome was unknown in three pneumonia patients.

^*e*^ The survival status at day 28 is missing in one patient belonging to group B.

**Fig 3:**
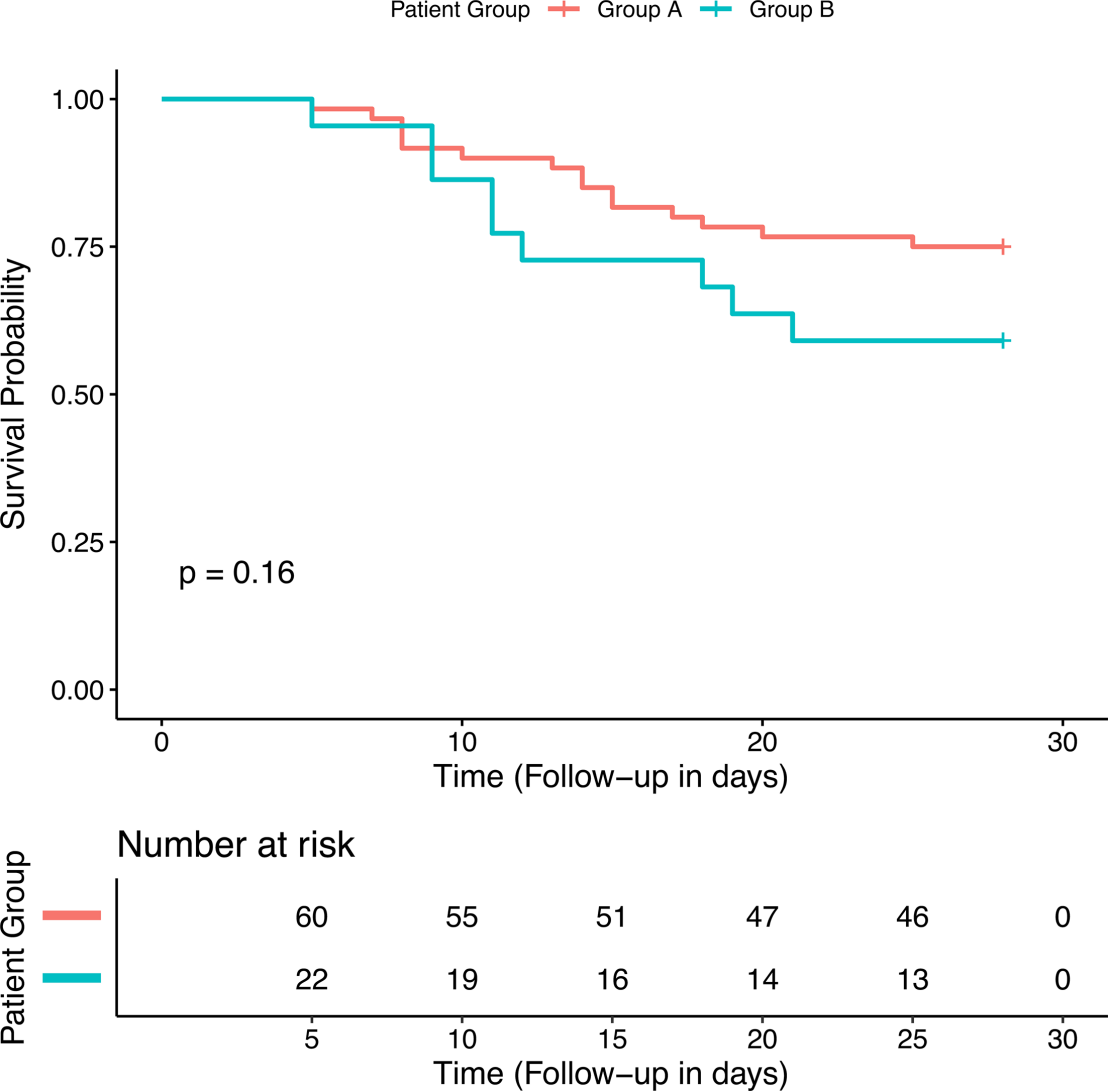
Survival at 28 days among 82 patients with pan-drug-resistant *A. baumannii* infections. Patients belonging to group A received definitive treatment with colistin, ampicillin-sulbactam, and meropenem, while group B patients received definitive treatment with colistin, ampicillin-sulbactam, and tigecycline.

In the unadjusted analyses, there was no statistically significant difference in survival on day 28 between the groups (*P*: 0.16, Fig. 3). The univariable analyses with the respective hazard ratios appear in Table S6 in the supplement. The same variables as for the primary outcome were introduced in a Cox regression model (Table 5). Regimen B treatment and SOFA score were significant predictors of 28-day mortality. After propensity matching, SOFA score and Charlson comorbidity index were statistically significant predictors, whereas treatment regimen had a borderline association (*P*: 0.051).

**TABLE 5:** Multivariable Cox regression analysis of factors associated with 28-day mortality^*a*^

Variable	Hazard ratio (95% CI)	*P* value
Before propensity matching		
Age	1.024 (0.982–1.067)	0.265
Charlson comorbidity index	1.123 (0.936–1.349)	0.212
SOFA score	1.219 (1.120–1.327)	<0.001
Regimen B treatment	2.528 (1.077–5.936)	0.033
After propensity matching		
Age	1.033 (0.978–1.090)	0.244
Charlson comorbidity index	1.224 (1.039–1.443)	0.016
SOFA score	1.248 (1.140–1.366)	<0.001
Regimen B treatment	2.641 (0.994–7.016)	0.051

^*a*^ The unadjusted model refers to the observed data. Propensity matching was performed via inverse probability of treatment weighting, where the probability of receiving regimen B was modelled as a function of patient disposition, infection type, and SOFA score at infection onset.

**Fig 4:**
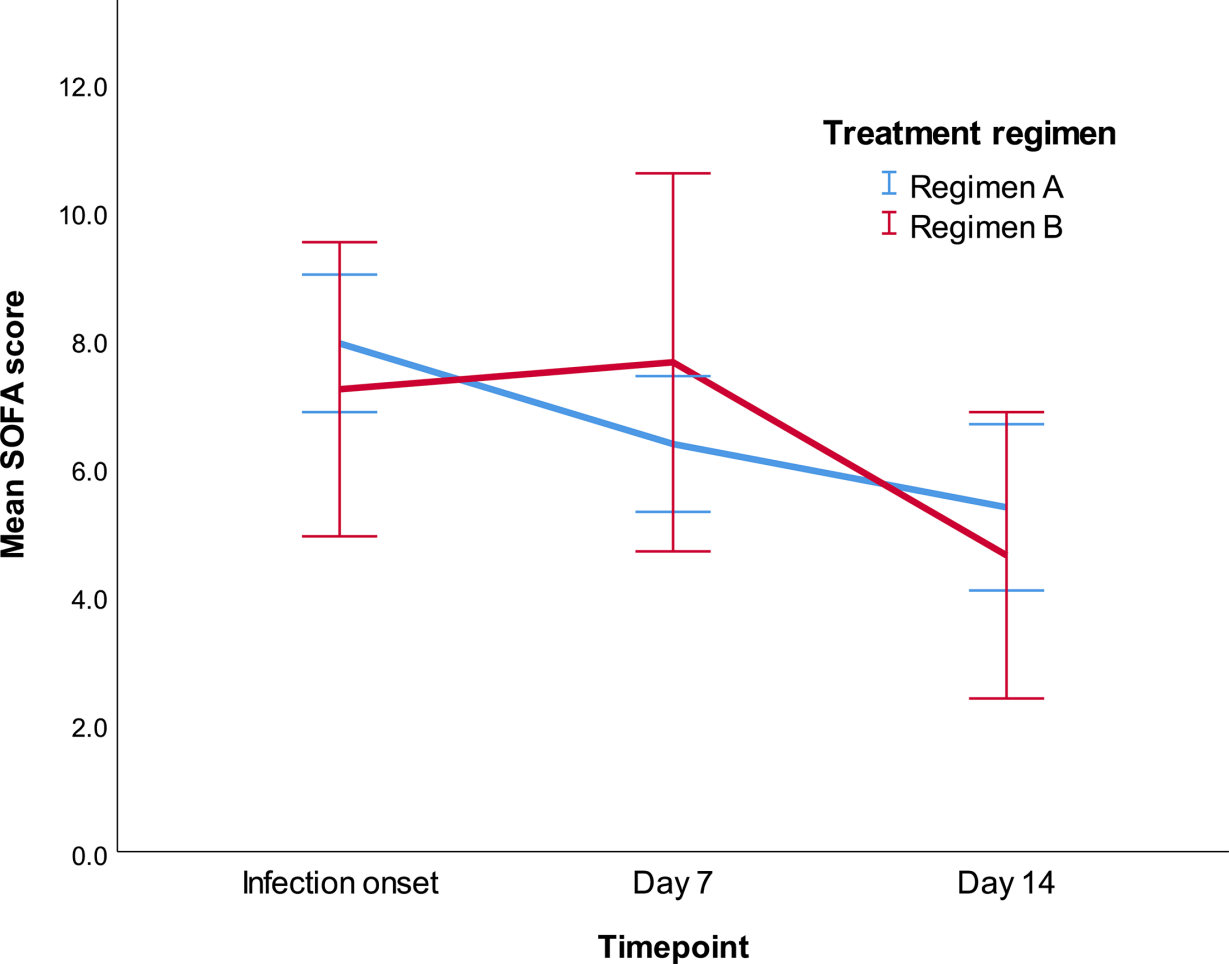
Resolution of multi-organ failure among survivors in the two treatment groups, as represented by SOFA scores measured on infection onset, day 7, and day 14. Error bars represent 95% confidence intervals.

The resolution of multi-organ failure by days 7 and 14 followed a different trajectory in the two groups. While treatment group *per se* was not a determinant of SOFA score at different time points (*P*: 0.635 for the effect of patient group on SOFA score), the interaction between time and patient group was statistically significant (*P*: 0.03) signifying different recovery patterns. In group A, there was a trend (*P*: 0.20) for a reduction in mean SOFA score of 1.57 (−0.49–3.63) points between infection onset and day 7 and a significant (*P*: 0.01) reduction of 2.56 (0.43–4.69) points between infection onset and day 14. In group B, there existed a statistically non-significant (*P*: 1.00) increase of 0.42 (−2.92–3.76) points and a non-statistically significant (*P*: 0.26) reduction of 2.59 points (−1.03–6.24), respectively (Fig. 4). Regarding safety, significantly more antimicrobial agent withdrawals/regimen adjustments were observed in group B patients, who also experienced a greater number of adverse events (Table S7). Collectively, the results suggest that Regimen A was associated with reduced chances of treatment failure, earlier and better physiology recovery, fewer adverse events, and a trend toward improved microbiological and survival outcomes.

## DISCUSSION

In this retrospective analysis of patients with severe PDR-AB infections, treatment with meropenem, ampicillin-sulbactam, and colistin was associated with a greater than threefold increase in the odds of successful treatment, compared with a combination of tigecycline, ampicillin-sulbactam, and colistin after adjusting for confounders and baseline imbalances. This finding persisted in three post hoc sensitivity analyses. Furthermore, a sizable difference in 28-day mortality was also noted. This difference was statistically significant in the multivariable analysis of the observed data and marginally fell off statistical significance after propensity matching.

The reason behind this unexpected difference is worth exploring. Our data suggest different trajectories in the resolution of multiorgan failure in the two groups, especially in the first days of treatment, where the mean SOFA scores trended in opposite directions. This divergence may reflect the superior bactericidal activity of the meropenem-based regimen. Supporting this hypothesis, Lenhard et al., using a hollow-fiber infection model, demonstrated that only the triple combination of polymyxin B, ampicillin-sulbactam, and meropenem effectively eradicated colistin-resistant CRAB bacterial populations, while monotherapy or two-drug regimens failed to do so (17). These findings, in conjunction with our clinical results, suggest a pharmacodynamic synergy among the agents in regimen A. Colistin disrupts the bacterial outer membrane, thereby facilitating the intracellular uptake of co-administered agents (18). Additionally, the efficacy of sulbactam, which exhibits activity against *A. baumannii* through high-affinity binding to penicillin-binding proteins (PBPs) 1 and 3, may be enhanced by meropenem, which primarily targets PBP2 (enhancer effect) (19–21). On the other hand, although it has been studied less extensively, it seems that tigecycline is much less likely to achieve synergy, as Karakonstantis et al. concluded in a systematic review aimed at summarizing the preclinical evidence for treating PDR-AB infections (22).

The components of the composite outcome of clinical failure by day 14 varied between treatment groups and may help explain observed differences. Apart from treatment modifications due to lack of efficacy, drug discontinuations due to perceived toxicity were notably more frequent in group B. Since the only differing agent between the regimens was tigecycline (group B) versus meropenem (group A), this implicates tigecycline as a potential contributor. In fact, tigecycline was withdrawn in five (22%) patients in group B due to suspected toxicity, whereas no patients in group A discontinued meropenem. These findings are consistent with prior reports linking tigecycline to increased adverse events and warnings of increases in mortality in critically ill patients (23). Nevertheless, when toxicity-related drug withdrawal was excluded from the definition of clinical failure, its effect on treatmentoutcome association was negligible, suggesting that toxicity was frequently superimposed on reduced efficacy.

Our results replicate the findings of previous research on multidrug-resistant (MDR) *A. baumannii* infections in the PDR-AB setting. Cheng et al. compared colistin-carbapenem with colistin-tigecycline in a prospective multicenter study of 55 patients with XDR*-*AB BSIs and concluded that the former regimen was associated with lower 14-day mortality (24). Similarly, tigecycline-based therapy was found to be inferior to colistin-based therapy in terms of mortality, in a matched cohort study of critically ill patients with MDR-AB infections, especially when the tigecycline minimum inhibitory concentrations were over 2 mg/L (25).

Combination therapy for *A. baumannii* infections has yielded conflicting results. Colistin with meropenem was ineffective in two randomized multicenter controlled trials, where *A. baumannii* accounted for the vast majority of infections (11, 12), yet the combination with sulbactam might be more promising (26, 27). Triple drug combinations are even less studied and almost not at all in a controlled manner. Apart from the Qureshi et al. (13) and Assimakopoulos et al. (14) studies, Heil et al. studied 18 patients with CRAB infections and concluded that the early initiation of polymyxin B, ampicillin-sulbactam, and meropenem is associated with favorable clinical courses (28).

The preference for Regimen A, suggested by our own data, is especially relevant for low- and middle-income countries, or regions where novel antimicrobial agents, such as cefiderocol and sulbactam-durlobactam, are not available or approved (29–31). Despite theoretical advantages, novel agents are not without controversy. Cefiderocol has raised concerns since the CREBIBLE-CR study demonstrated increased 28-day mortality in cefiderocol-treated patients, compared to best available therapy (BAT), particularly in CRAB infections (32). Similarly, sulbactam-durlobactam has shown promise in the ATTACK trial, in terms of safety and improved survival compared to colistin (33). However, as of now, it remains available only in the US, limiting its clinical utility in broader global practice. Additionally, neither cefiderocol nor sulbactam/durlobactam has been directly compared to alternative BAT based on high-dose ampicillin/sulbactam (34). Eravacycline exhibits lower MICs than tigecycline and maintains activity against resistant *A. baumannii.* However, it was associated with worse clinical outcomes, compared to BAT, in a recent retrospective study (35).

The size of our population can be considered a strength of our study. Previous publications on PDR-AB infections were single- or two-center studies, with low numbers of patients. Furthermore, our study was controlled and can thus provide evidence to inform clinical decisions. Third, suboptimal antimicrobial dosing has been recognized as a limitation of published studies (12). Reflecting HSCH guidance and usual practice in Greece, antimicrobial doses were high and concordant with international recommendations. Our study is not without limitations. Due to the observational nature of the study, we cannot exclude the presence of hidden confounders, especially since regimen selection was based on treating physicians’ preferences. Nevertheless, the two groups were relatively balanced by themselves, and furthermore, we tried to absorb differences via propensity matching. Second, the number of clinical failure events was relatively small, especially in group B, which is reflected in the wide confidence intervals around our estimates. Third, the pathogens were not studied in a central laboratory, and in a small minority, colistin susceptibility was not evaluated with broth microdilution.

### Conclusion

Treatment of pan-drug-resistant *A. baumannii* infections with colistin, ampicillin-sulbactam, and meropenem was associated with improved outcomes compared to a regimen containing colistin, ampicillin-sulbactam, and tigecycline. This study supports the use of the former regimen as an effective, safe, accessible, and affordable solution for the treatment of PDR-AB infections.

## MATERIALS AND METHODS

### Setting and patient population

DESPAIR is a prospective, multicenter study of HSCH, collecting data on severe CRAB infections from 12 sites in 11 tertiary care hospitals throughout Greece. Herein, we present a retrospective analysis of the data set containing information on PDR-AB infections treated with one of two previously described triple-drug combinations: high-dose ampicillin-sulbactam, colistin, and meropenem (regimen A), or high-dose ampicillin-sulbactam, colistin, and tigecycline (regimen B). Chronologically, the first patient was enrolled in February 2022, and the last in June 2024. Patients enrolled in the study had to have received either regimen, based on a decision made by treating physicians, within 96 h of the onset of infection, as a definitive treatment for a BSI or hospital-acquired pneumonia (HAP; including cases of ventilated HAP [vHAP] and VAP) caused by PDR-AB. The doses of antimicrobials were in accordance with HSCH guidance (36) (Table S2).

At least one positive blood culture or one positive quantitative bronchial secretion culture, using standard thresholds (37), for PDR-AB was required for inclusion of BSI and pneumonia patients, respectively. Patients under concurrent Gram-positive or nebulized treatment were excluded. Infections had to be monomicrobial, and only the initial episode of PDR-AB infection was considered. Patients who died within 48 h of commencing treatment with study regimens were included in the study but excluded from the outcome analysis.

Pertinent information, including demographic characteristics, underlying diseases, Charlson comorbidity index, clinical and laboratory infection markers, APACHE II score on admission, and the SOFA score at infection onset and during treatment, was recorded in an electronic case report form. Study data were collected and managed using REDCap electronic data capture tools hosted at the University of Athens, Greece. Vital status and patient disposition were recorded on day 28, along with critical care resource utilization and organ support (if relevant) up to day 28.

### Microbiology

Species identification and susceptibility testing were performed using standard procedures and automated systems at each site’s clinical microbiology laboratory. Sites were encouraged to use broth microdilution for the determination of susceptibility to colistin according to European Committee of Antimicrobial Susceptibility Testing (EUCAST) recommendations. Results were interpreted according to the 2025 EUCAST clinical breakpoints (v.15.0). For ampicillin-sulbactam and tigecycline, isolates with MICs greater than 8 and 2 mg/L, respectively, were considered resistant. An isolate was characterized as PDR if it was resistant to all available antimicrobials (10); cefiderocol and sulbactam-durlobactam were not available, and they were not tested.

### Outcome

The primary outcome was the rate of clinical failure by day 14 from infection onset. Secondary outcomes included the individual components of clinical failure, all-cause 28-day mortality, microbiological eradication in the microbiologically evaluable population on day 14, the evolution of SOFA score among survivors, the proportion of patients with relapse, superinfection, or adverse events, and the intervention-free days up to day 28.

### Definitions

Standard definitions were employed for hospital-acquired BSI and pneumonia (38). Sepsis and septic shock were defined according to Sepsis 3 definitions (39). The underlying illnesses were classified as rapidly fatal, ultimately fatal, and non-fatal according to the modified McCabe-Jackson classification (15). Infection onset was defined as the calendar day when the index culture yielding PDR-AB was taken. Patients with one of the following were considered immunosuppressed: neutropenia (<1,000 cells/μL at any time during current admission in the context of hematological disease or receipt of myelosuppressive treatment), corticosteroid administration at a daily dose of at least 15 mg prednisolone (or equivalent) for at least 20 days during the previous 3 months, history of bone marrow or solid organ transplantation, or HIV infection with CD4 cell count below 200/μL at steady state before the index infection. Antimicrobial therapy given before obtaining the susceptibility results was defined as empirical, and treatment after the susceptibility testing had become available was defined as definitive.

Clinical failure at day 14 was defined as meeting any of the following: (i) death before day 14, (ii) persisting bacteremia after day 5 for BSI patients (i.e., failure to “clear” blood pathogen within 5 days from infection onset), (iii) need for salvage antimicrobial therapy (further addition of antibiotics or substitution of a study regimen agent) due to perceived clinical deterioration as judged by treating physicians, (iv) withdrawal of one or more constituents of study regimens due to toxicity, as judged by treating physicians, and (v) absence of oxygenation improvement in pneumonia patients. For VAP or vHAP patients, we defined oxygenation improvement as either being liberated from mechanical ventilation or an increase in the PaO_2_/FiO_2_ ratio of at least 100. For HAP patients, oxygenation improvement was defined as the non-progression to mechanical ventilation until day 14.

Microbiological cure was only assessed in patients with repeat specimens obtained from the respective sites of infection between days 6 and 14. If the repeat culture was negative, this was defined as eradication of the pathogen from the infection site. In the absence of clinical reasons to repeat cultures, eradication was considered “presumed.”

The reappearance of signs and symptoms of infection during follow-up after the end of the treatment period and up to day 28 from infection onset was defined as a relapse if caused by the same pathogen and as a superinfection if caused by a different organism.

### Statistical analyses

Typical descriptive statistics were employed. Continuous variables are summarized as means and standard deviations or as medians and interquartile ranges, based on their distribution. Group comparisons for continuous variables were performed with the Student’s *t*-test or the Mann-Whitney U-test, as appropriate. Categorical variables are presented as counts and proportions, and between-group comparisons were done with χ^2^ or Fisher’s exact tests, as appropriate.

The effects of collected variables on the primary outcome were assessed with univariable and multivariable logistic regression analyses. Odds ratios (ORs) and 95% confidence intervals (CIs) were computed. Variables were introduced in the multivariable model based on their clinical significance and statistical association with clinical failure. The treatment regimen was forced into the model. The goodness-of-fit of the constructed models was assessed via the Akaike Information Criterion.

To adjust for baseline differences between the two groups, a propensity score for receiving regimen B was calculated via logistic regression, where infection type (pneumonia vs BSI), patient disposition (ICU vs ward), and infection onset SOFA score were entered as covariates (Table S8). The probability of receiving regimen B was used to construct inverse probability of treatment weighted cohorts. Standardized mean differences before and after adjustment appear in Tables S1 and S9. As a primary analysis, an adjusted logistic regression model with robust standard errors (Huber-White) was fitted in the IPTW cohort. Three post hoc sensitivity analyses were performed. In the first, variable selection was based solely on their statistical association with the primary outcome. In the second, propensity matching was not performed via IPTW but with the introduction of the probability of receiving treatment B as a covariate in the logistic regression model. The third analysis sought to decipher the impact on our results of drug toxicity as a cause of clinical failure. Herein, we used a different composite outcome where clinical failure was defined as either death by day 14, need for rescue therapy for the index infection and bacteremia persistence for BSI patients, or failure to improve in oxygenation or progression to intubation for pneumonia patients.

Among secondary outcomes, unadjusted survival analyses were performed with the Kaplan-Meier method and the two groups were compared with the log-rank test. The effect of treatment regimen on 28-day mortality was evaluated with univariable and multivariable Cox regression analyses. Variable selection was based on the same principles as for the primary outcome. Adjusted analyses were also performed in the IPTW cohort. The evolution of multiorgan dysfunction up to day 14 in the two treatment groups was assessed with a linear mixed model where SOFA score (measured on infection onset and days 7 and 14) was the dependent variable and time, treatment group, and their interaction were covariates. Resource utilization indices were calculated by summing the days the respective modality was used up to day 28. To adjust for death being a competing event, the value of −1 was assigned to intervention-free days in patients in whom death was the final outcome, as proposed by Angus et al. (40)

There was no imputation of missing data. All analyses were done with the intention to treat principle and were performed with IBM SPSS Statistics version 26 and RStudio version 2024.12.1. An α-level of 0.05 was used for statistical significance.

## SUPPLEMENTAL MATERIAL

Supplemental material aac.01154-25-s0001.docx Tables S1 to S9; Fig. S1 and S2.

ASM does not own the copyrights to Supplemental Material that may be linked to, or accessed through, an article. The authors have granted ASM a non-exclusive, world-wide license to publish the Supplemental Material files. Please contact the corresponding author directly for reuse.
