# Residual confounding and outcome definition in observational comparisons of PDR-*Acinetobacter baumannii* therapies

**DOI:** 10.1128/aac.00084-26

**Published:** 2026-03-16

**Authors:** Carsten Külls, Niloufar Dadashpour, Majid Golestanieraghi

**Affiliations:** 1 Department of Anaesthesiology and Critical Care, Helios Klinikum Schleswig 440814 , Schleswig, Germany; Houston Methodist Hospital and Weill Cornell Medical College, Houston, Texas, USA

**Keywords:** observational studies, treatment outcome, antibacterial agents, combination therapy, *Acinetobacter baumannii*, drug resistance

## LETTER

We read with interest the DESPAIR study by Pontikis et al., comparing two triple-antimicrobial regimens for pan–drug-resistant *Acinetobacter baumannii* (PDR-AB) infections (1). While the clinical question is important, several methodological limitations preclude any definitive inference regarding regimen superiority.

First, despite the use of inverse probability weighting, the analysis remains vulnerable to residual confounding. Although global severity indices such as the Charlson comorbidity index (median 5 vs 4, *P* = 0.803) and SOFA score at infection onset (median 8 vs 7, *P* = 0.272) were well balanced, major determinants of respiratory severity were not. Patients receiving the meropenem-based regimen had substantially higher rates of ICU admission (93% vs 68%, *P* = 0.002), invasive mechanical ventilation (80% vs 56%, *P* = 0.021), and ventilator-associated pneumonia (85% vs 53%, *P* = 0.018). These variables are strongly associated with mortality and treatment failure in *A. baumannii* pneumonia and are unlikely to be fully captured by SOFA alone. Their omission from the propensity model raises concern that residual confounding, rather than treatment effect, may explain the observed associations.

Second, the composite primary outcome is driven largely by subjective, clinician-dependent components. Treatment discontinuation due to toxicity occurred in 26% of patients receiving the tigecycline-based regimen compared with 1.7% in the comparator group, with 5 of 23 patients having tigecycline withdrawn explicitly because of suspected drug-related toxicity. In an unblinded observational study, such decisions are highly susceptible to performance and attribution bias, particularly for agents with a recognized adverse-event profile (2). Consequently, the composite endpoint risks reflecting differential clinician behavior rather than intrinsic antimicrobial efficacy.

Third, the relationship between clinical failure and mortality is inconsistent. Despite an odds ratio of approximately 3.8 for clinical failure after weighting, unadjusted 28-day mortality was numerically higher but not statistically different in the tigecycline group (41% vs 25%, *P* = 0.161), and the adjusted hazard ratio remained borderline (2.64, 95% CI = 0.99–7.02). This discordance suggests that the observed treatment effect is fragile and highly dependent on the chosen endpoint and modeling strategy.

Fourth, extrapolation from preclinical synergy data (3) overlooks important clinical contradictions. In a randomized controlled trial, colistin plus meropenem was inferior to colistin monotherapy for severe carbapenem-resistant gram-negative infections dominated by *A. baumannii* (4). Conversely, observational data have shown favorable outcomes with colistin–sulbactam combinations—without meropenem—reporting mortality as low as 18% in extensively drug-resistant *A. baumannii* infections (5). These findings suggest that host severity and source control may outweigh incremental differences between combination regimens (6, 7).

Finally, interpretation of toxicity signals is complicated by the shared exposure to colistin in both regimens. Polymyxin-associated nephrotoxicity is well established and may interact with concomitant agents, further confounding attribution of adverse events to individual drugs in combination therapy (8).

In conclusion, although the DESPAIR study contributes valuable observational data, its methodological limitations prevent any robust conclusion regarding the superiority of one triple-drug regimen over another. The available evidence cannot support preferential adoption of the meropenem-based regimen, and the conclusions should be interpreted with appropriate caution.
