# Supplementary material for: Comparative effectiveness of two antimicrobial regimens for the treatment of pan-drug-resistant Acinetobacter baumannii infections: results from the DESPAIR study
Source: Antimicrob Agents Chemother. 2026 Jan 15;70(3):e01154-25. doi: 10.1128/aac.01154-25 (PMC12959155; doi:10.1128/aac.01154-25)
Supplement: Supplemental material — Tables S1 to S9; Fig. S1 and S2. [file aac.01154-25-s0001.docx]

# Supplementary Tables

|  | **Group A (colistin, ampicillin-sulbactam, meropenem)** | **Group B (colistin, ampicillin-sulbactam, tigecycline)** | **p-value** | **SMD** |
| --- | --- | --- | --- | --- |
| n | 61 | 25 |  |  |
| Age | 66.89 (15.97) | 67.84 (11.36) | 0.787 | 0.069 |
| Female gender | 23 (37.7) | 12 (48.0) | 0.522 | 0.209 |
| Charlson comorbidity index | 4.84 (2.94) | 4.88 (3.07) | 0.951 | 0.015 |
| McCabe-Jackson classification |  |  | 0.574 | 0.245 |
| Non-fatal disease | 48 (78.7) | 17 (68.0) |  |  |
| Ultimately fatal disease | 10 (16.4) | 6 (24.0) |  |  |
| Rapidly fatal disease | 3 (4.9) | 2 (8.0) |  |  |
| COVID-19 | 12 (19.7) | 2 (8.0) | 0.313 | 0.343 |
| Respiratory reason for ICU admission | 44 (75.9) | 10 (55.6) | 0.173 | 0.438 |
| Cardiovascular reason for ICU admission | 30 (51.7) | 7 (38.9) | 0.495 | 0.260 |
| Chronic kidney disease | 9 (14.8) | 2 (8.0) | 0.620 | 0.214 |
| Chronic heart failure | 9 (14.8) | 5 (20.0) | 0.782 | 0.139 |
| Coronary heart disease | 11 (18.0) | 7 (28.0) | 0.459 | 0.238 |
| Chronic obstructive pulmonary disease | 18 (29.5) | 5 (20.0) | 0.525 | 0.222 |
| Admission type |  |  | 0.124 | 0.603 |
| Medical | 47 (77.0) | 17 (68.0) |  |  |
| Urgent surgical | 5 (8.2) | 6 (24.0) |  |  |
| APACHE II score | 20.70 (7.62) | 20.38 (8.41) | 0.862 | 0.041 |
| Time from infection onset to sampling (days) | 0.77 (0.97) | 1.04 (1.27) | 0.291 | 0.238 |
| SOFA score | 8.00 (4.16) | 7.16 (5.14) | 0.430 | 0.180 |
| Pneumonia as infection type | 47 (77.0) | 17 (76.0) | 1.000 | 0.025 |
| Pneumonia type |  |  | 0.018 | 0.758 |
| VAP | 40 (85.1) | 10 (52.6) |  |  |
| ventilated HAP | 3 (6.4) | 5 (26.3) |  |  |
| HAP | 4 (8.5) | 4 (21.1) |  |  |
| Bacteremic pneumonia | 27 (57.4) | 12 (63.2) | 0.880 | 0.117 |
| Time from infection onset to AST report (days) | 2.46 (1.25) | 2.56 (1.58) | 0.754 | 0.071 |
| Infection onset maximum temperature (◦C) | 37.80 (1.00) | 37.92 (0.82) | 0.586 | 0.135 |
| Infection onset lower SAP (mmHg) | 100.64 (20.77) | 102.40 (18.17) | 0.713 | 0.090 |
| Infection onset higher heart rate (bpm) | 103.74 (17.75) | 98.20 (19.65) | 0.206 | 0.296 |
| Infection onset higher respiratory rate (bpm) | 23.51 (5.75) | 23.20 (4.92) | 0.815 | 0.058 |
| Infection onset vasopressor administration | 39 (63.9) | 12 (48.0) | 0.261 | 0.325 |
| IMV at infection onset | 49 (80.3) | 14 (56.0) | 0.041 | 0.541 |
| CRRT at infection onset | 10 (16.4) | 2 (8.0) | 0.498 | 0.259 |
| Lactate (mEq/L) | 1.62 (1.15) | 1.74 (2.16) | 0.755 | 0.067 |
| White blood cells (/μL) | 14.64 (7.05) | 13.33 (6.65) | 0.430 | 0.191 |
| Active empiric antimicrobials per patient | 0 (0) | 0 (0) | N/A | N/A |
| Ward (vs ICU) as the patient disposition | 4 (6.6) | 8 (32.0) | 0.006 | 0.681 |
| Sepsis classification |  |  | 0.124 | 0.464 |
| No sepsis | 8 (13.1) | 8 (32.0) |  |  |
| Sepsis | 28 (45.9) | 9 (36.0) |  |  |
| Septic shock | 25 (41.0) | 8 (32.0) |  |  |

### Table S1. Standardized mean differences between treatment groups before the application of inverse probability of treatment weights. Categorical variables are presented as n (%). Numerical variables are presented as mean (SD). COVID-19: Coronavirus Disease 19, ICU: Intensive Care Unit, APACHE: Acute Physiology And Chronic Health Evaluation, SOFA: Sepsis-related Organ Function Assessment, VAP: Ventilator-Associated Pneumonia, HAP: Hospital-Acquired Pneumonia, AST: Antimicrobial Susceptibility Testing, SAP: Systolic Arterial Pressure, bpm: beats/breaths per minute, IMV: Invasive Mechanical Ventilation, CRRT: Continuous Renal Replacement Therapy.

| Meropenem dosage | | |
| --- | --- | --- |
| Creatinine clearance (ml/min) | Suggested dose (gr) | Frequency |
| ≥ 50 | 2 | q8h |
| 25-49 | 2 | q12h |
| 10-24 | 1 | q12h |
| <10 | 1 | q24h |
| CRRT | 1 | q8h |
|  | | |
| Ampicillin-sulbactam dosage | | |
| Creatinine clearance (ml/min) | Suggested dose (gr) | Frequency |
| ≥ 50 | 9 | q8h |
| 30-49 | 6 | q8h |
| 7-29 | 6 | q12h |
| CRRT | 6 | q12h |
|  | | |
| Colistin dosage (maintenance dose; after a 9 million IU loading dose, irrespective of renal function; 1^st^ maintenance dose at 12 hours post loading dose) | | |
| Creatinine clearance | Suggested dose (million IU) | Frequency |
| ≥ 90 | 5.45 | q12h |
| 80 – 89 | 5.15 | q12h |
| 70 – 79 | 4.5 | q12h |
| 60 – 69 | 4.2 | q12h |
| 50 – 59 | 3.7 | q12h |
| 40 – 49 | 3.35 | q12h |
| 30 – 39 | 3 | q12h |
| 20 – 29 | 2.65 | q12h |
| 10 – 19 | 2.45 | q12h |
| 5 – 9 | 2.2 | q12h |
| < 5 | 2 | q12h |
| CRRT | 4 – 6.5 | q12h |
|  | | |
| Tigecycline dosage | | |
| Creatinine clearance (ml/min) | Suggested dose (mg) | Frequency |
| All values | 100 | q12h |

### Table S2. Suggested dosing regimens for antimicrobials under study. CRRT: continuous renal replacement therapy

|  | **Odds ratio (95% CI)** | **p-value** |
| --- | --- | --- |
| Regimen B treatment | 6.041 (1.563-27.970) | 0.013 |
| APACHE II score | 1.163 (1.055-1.305) | 0.005 |
| PaCO2 | 1.082 (1.028-1.149) | 0.005 |
| Creatinine | 1.454 (0.846-2.715) | 0.195 |

### Table S3. First sensitivity analysis. Logistic regression model for the prediction of clinical failure on day 14. Apart from regimen B treatment which was the variable of interest, variable selection was based on statistical criteria and their association with the dependent variable in univariate analyses. This model had the lowest Akaike Information Criterion (AIC) value (81.972). For comparison, the selected primary model had an AIC value of 108.670. APACHE II score was referenced to the ICU admission for ICU patients and ward admission for ward patients and distanced, on a median, 8.0 and 7.0 days from infection onset for groups A and B, respectively. PaCO2 and serum creatinine are referenced to infection onset. CI: Confidence Intervals, APACHE: Acute Physiology And Chronic Health Evaluation, PaCO2: Partial Pressure of Carbon Dioxide in the arterial blood. Regimen B consisted of Colistin, Ampicillin-Sulbactam and Tigecycline and is compared to Regimen A which consisted of Colistin, Ampicillin-Sulbactam and Meropenem.

| **Variable** | **Odds ratio (95% CI)** | **p-value** |
| --- | --- | --- |
| Regimen B treatment | 3.772 (1.183-13.178) | 0.029 |
| Infection onset SOFA score | 1.100 (0.987-1.236) | 0.093 |
| Charlson comorbidity index | 1.051 (0.840-1.310) | 0.657 |
| Age | 1.019 (0.974-1.070) | 0.427 |
| Probability of receiving regimen B | 0.374 (0.020-5.544) | 0.485 |

### Table S4. Second Sensitivity Analysis. Logistic regression model for the prediction of clinical failure by day 14. In this analysis, matching was not performed via inverse probability of treatment weighting but with introducing the probability of receiving regimen B into the model. Regimen B consisted of Colistin, Ampicillin-Sulbactam and Tigecycline and is compared with Regimen A which consisted of Colistin, Ampicillin-Sulbactam and Meropenem. SOFA: Sepsis-related Organ Function Assessment Score.

| **Before propensity matching** | | |
| --- | --- | --- |
| **Variable** | **OR (95% CI)** | **p-value** |
| Regimen B treatment | 2.943 (1.019-8.856) | 0.048 |
| Age | 1.024 (0.977-1.078) | 0.345 |
| Charlson comorbidity index | 1.094 (0.873-1.369) | 0.429 |
| SOFA score | 1.116 (1.000-1.256) | 0.057 |
| **After propensity matching** | | |
| Regimen B treatment | 3.215 (1.034-10.001) | 0.044 |
| Age | 1.006 (0.950-1.065) | 0.840 |
| Charlson comorbidity index | 1.172 (0.889-1.543) | 0.260 |
| SOFA score | 1.079 (0.958-1.216) | 0.210 |

### Table S5.Third sensitivity analysis. Multivariable logistic regression analysis of a version of the primary outcome where toxicity-related drug withdrawal was excluded as a cause of clinical failure. The unadjusted model refers to the observed data. In the adjusted model, propensity matching was performed via inverse probability of treatment weighting, where the probability of receiving regimen B was modelled as a function of patient disposition, infection type and SOFA score at infection onset. Robust standard errors (Huber-White) were implemented for model stability. OR: Odds ratio, CI: Confidence Intervals, SOFA score: Sepsis-related Organ Function Assessment score.

|  | | | | **Died** | **Survived** | **Total** | **HR** | **p-value** |
| --- | --- | --- | --- | --- | --- | --- | --- | --- |
| n | | | | 24 | 58 | 82 | n/a | n/a |
| Treatment group B | | | | 9 (38) | 13 (22) | 22 (27) | 1.799 (0.787-4.113) | 0.164 |
| Treatment group A | | | | 15 (62) | 45 (78) | 60 (73) | 0.556 (0.243-1.271) | 0.164 |
| Age | | | | 75 (67-79) | 68 (57-76) | 70 (59-77) | 1.021 (0.989-1.054) | 0.193 |
| Male gender | | | | 14 (58) | 34 (58) | 48 (59) | 0.961 (0.427-2.163) | 0.923 |
| ICU stay (2) (vs ward (1)) | | | | 21 (88) | 50 (86) | 71 (87) | 0.917 (0.273-3.076) | 0.889 |
| COVID-19 | | | | 5 (21) | 9 (16) | 14 (17) | 1.373 (0.512-3.678) | 0.529 |
| Smoking | | | | 9 (45) | 30 (63) | 39 (57) | 0.554 (0.230-1.339) | 0.190 |
| Immunosuppression | | | | 3 (13) | 5 (9) | 8 (10) | 1.344 (0.401-4.507) | 0.632 |
| McCabe-Jackson | Non-lethal disease | | | 11 (46) | 51 (88) | 62 (76) | reference | <0.001 |
|  | Ultimately lethal disease | | | 12 (50) | 4 (7) | 16 (20) | 0.766 (0.099-5.937) | 0.799 |
|  | Rapidly fatal disease | | | 1 (4) | 3 (5) | 4 (5) | 4.293 (0.556-33.112) | 0.162 |
| Known colonization with XDR/PDR A.baumannii | | | | 1 (4) | 9 (16) | 10 (12) | 0.287 (0.039-2.127) | 0.222 |
| Diabetes | None | | | 16 (67) | 40 (69) | 56 (68) | reference | 0.768 |
|  | Uncomplicated | | | 3 (13) | 9 (16) | 12 (15) | 0.808 (0.235-2.774) | 0.735 |
|  | End-organ damage | | | 5 (21) | 9 (16) | 14 (17) | 1.333 (0.488-3.640) | 0.575 |
| Malignancy | None | | | 19 (79) | 49 (85) | 68 (83) | reference | 0.882 |
|  | Any leukemia/lymphoma or localized solid tumor | | | 4 (17) | 7 (12) | 11 (13) | 0.843 (0.113-6.302) | 0.868 |
|  | Metastatic solid | | | 1 (4) | 2 (3) | 3 (4) | 1.102 (0.123-9.865) | 0.931 |
| Congestive heart failure | | | | 6 (25) | 8 (14) | 14 (17) | 1.784 (0.708-4.499) | 0.220 |
| Chronic obstructive pulmonary disease | | | | 7 (29) | 16 (28) | 23 (28) | 1.078 (0.447-2.599) | 0.868 |
| Charlson comorbidity index | | | | 6.0 (4.0-7.5) | 4.0 (2.0-6.0) | 5.0 (3.0-6.0) | 1.130 (0.989-1.292) | 0.071 |
| APACHE II score, mean (SD) | | | | 24.8 (7.3) | 18.9 (6.9) | 20.6 (7.5) | 1.109 (1.044-1.179) | 0.001 |
| Time to microbiological confirmation | | | | 0.0 (0.0-1.0) | 0.5 (0.0-2.0) | 0.0 (0.0-1.0) | 0.868 (0.571-1.319) | 0.508 |
| Bloodstream infection (0) (vs pneumonia (1)) | | | | 7 (29) | 13 (22) | 20 (24) | 0.778 (0.323-1.878) | 0.577 |
| Bloodstream infection type | | | Primary | 6 (86) | 9 (69) | 15 (75) | reference |  |
|  |  |  | CRBSI | 1 (14) | 3 (23) | 4 (20) | N/A |  |
|  |  |  | Secondary | 0 (0) | 1 (8) | 1 (5) | N/A |  |
| Pneumonia type | | | VAP | 10 (59) | 31 (69) | 41 (66) | Reference | 0.178 |
|  |  |  | vHAP | 4 (24) | 3 (7) | 7 (11) | 1.157 (0.318-4.206) | 0.825 |
|  |  |  | HAP | 3 (18) | 11 (24) | 14 (23) | 3.216 (0.719-14.391) | 0.127 |
| Bacteremic pneumonia | | | | 11 (65) | 26 (58) | 37 (60) | 1.377 (0.509-3.724) | 0.529 |
| Time to AST reporting | | | | 2.5 (2.0-3.0) | 20. (2.0-3.0) | 20. (2.0-3.0) | 1.105 (0.804-1.518) | 0.539 |
| Sepsis level | | No sepsis (0) | | 3 (13) | 12 (21) | 15 (18) | reference | 0.014 |
|  |  | Sepsis (1) | | 6 (25) | 30 (52) | 36 (44) | 0.814 (0.204-3.256) | 0.771 |
|  |  | Septic shock (2) | | 15 (63) | 16(28) | 31 (38) | 2.989 (0.865-10.332) | 0.084 |
| Vasopressors | | | | 19 (79) | 30 (52) | 49 (60) | 2.931 (1.094-7.854) | 0.032 |
| Respiratory rate | | | | 24 (20-26) | 24 (18-26) | 24 (19-26) | 0.999 (0.932-1.072) | 0.981 |
| Highest temperature, mean (SD) | | | | 37.6 (1.0) | 38.0 (0.7) | 37.9 (0.8) | 0.599 (0.370-0.970) | 0.037 |
| Lowest systolic arterial pressure | | | | 105 (95-113) | 105 (94-115) | 105 (94-115) | 1.001 (0.981-1.021) | 0.926 |
| Highest heart rate, mean (SD) | | | | 101 (22) | 101 (17) | 101 (18) | 1.001 (0.979-1.024) | 0.927 |
| Invasive mechanical ventilation | | | | 20 (83) | 40 (69) | 60 (73) | 1.976 (0.675-5.783) | 0.214 |
| Continuous Renal Replacement Therapy | | | | 7 (29) | 4 (7) | 11 (13) | 3.826 (1.577-9.284) | 0.003 |
| PaO2 | | | | 84 (75-95) | 81 (72-98) | 82 (73-97) | 1.006 (0.989-1.024) | 0.492 |
| PaCO2 | | | | 42 (35-53) | 41 (35-49) | 41 (35-49) | 1.009 (0.974-1.046) | 0.614 |
| pH | | | | 7.40 (7.30-7.44) | 7.44 (7.38-7.48) | 7.42 (7.35-7.47) | 0.000 (0.000-0.057) | 0.002 |
| Lactate | | | | 2.0 (1.3-2.5) | 1.1 (0.9-1.6) | 1.4 (1.0-1.8) | 1.355 (1.152-1.593) | <0.001 |
| Urea | | | | 91 (68-152) | 47 (30-86) | 64 (38-100) | 1.013 (1.007-1.019) | <0.001 |
| Creatinine | | | | 1.5 (0.9-2.7) | 0.8 (0.6-1.2) | 0.9 (0.6-1.4) | 1.642 (1.281-2.104) | <0.001 |
| Bilirubin | | | | 0.9 (0.6-1.8) | 0.6 (0.4-0.8) | 0.7 (0.5-1.1) | 1.066 (1.011-1.124) | 0.018 |
| Aspartate aminotransferase | | | | 35 (25-78) | 36 (25-57) | 35 (25-61) | 1.001 (1.000-1.002) | 0.001 |
| Alanine aminotransferase | | | | 39 (26-66) | 37 (18-53) | 37 (21-59) | 1.001 (1.000-1.002) | 0.015 |
| Albumin, mean (SD) | | | | 2.8 (0.5) | 2.9 (0.6) | 2.9 (0.5) | 0.700 (0.306-1.601) | 0.398 |
| Prothrombin time | | | | 13.0 (11.6-14.0) | 12.3 (11.3-14.3) | 12.8 (11.5-14.3) | 1.149 (0.994-1.330) | 0.061 |
| Activated Partial Thromboplastin Time | | | | 38 (34-41) | 33 (30-37) | 34 (31-38) | 1.049 (1.009-1.091) | 0.017 |
| Hemoglobin | | | | 9.5 (8.4-11.2) | 9.2 (8.5-10.7) | 9.2 (8.4-10.8) | 1.087 (0.955-1.238) | 0.206 |
| Platelets | | | | 197 (72-264) | 251 (169-360) | 242 (150-332) | 0.996 (0.992-0.999) | 0.019 |
| White Blood Cells | | | | 12.6 (10.2-19.9) | 13.2 (7.8-17.0) | 12.9 (9.2-17.7) | 1.026 (0.974-1.082) | 0.335 |
| Neutrophils (%) | | | | 87 (80-93) | 84 (77-89) | 86 (79-91) | 1.042 (0.987-1.100) | 0.137 |
| Respiratory SOFA | | | | 2.5 (1.0) | 2.2 (1.0) | 2.3 (1.0) | 1.336 (0.849-2.100) | 0.210 |
| Hemodynamic SOFA | | | | 2.7 (1.6) | 1.8 (1.8) | 2.1 (1.7) | 1.331 (1.024-1.730) | 0.032 |
| Liver SOFA, mean (SD) | | | | 0.8 (1.3) | 0.1 (0.4) | 0.3 (0.8) | 1.754 (1.310-2.347) | <0.001 |
| Coagulation SOFA, mean (SD) | | | | 0.8 (1.4) | 0.3 (0.6) | 0.4 (0.9) | 1.547 (1.121-2.133) | 0.008 |
| Brain SOFA, mean (SD) | | | | 2.3 (1.6) | 1.6 (1.4) | 1.8 (1.5) | 1.290 (0.981-1.696) | 0.069 |
| Renal SOFA, mean (SD) | | | | 1.5 (1.5) | 0.5 (1.0) | 0.8 (1.2) | 1.585 (1.227-2.048) | <0.001 |
| SOFA score | | | | 10.5 (7.5-13.0) | 6.0 (4.0-9.0) | 8.0 (4.0-10.0) | 1.172 (1.086-1.265) | <0.001 |
| Empiric treatment receipt | | | | 26 (96) | 49 (85) | 72 (88) | 3.567 (0.482-26.415) | 0.213 |
| Active empiric regimen | | | | 0 (0) | 0 (0) | 0 (0) | N/A | N/A |
| Number of active empiric antibiotics | | | | 0 (0) | 0 (0) | 0 (0) | N/A | N/A |

### Table S6. Univariate Cox regression analysis of day 28 mortality. Categorical variables are expressed as counts and proportions, n (%). Numerical variables are expressed as medians (interquartile range), unless stated otherwise. Treatment group A consisted of colistin, ampicillin-sulbactam and meropenem while treatment group B consisted of colistin, ampicillin-sulbactam and tigecycline. ICU: intensive care unit, COVID-19: coronavirus-19 disease, XDR: extensively Drug-Resistant, PDR: Pan-Drug Resistant, APACHE: Acute Health and Chronic Health Evaluation, CRBSI: Catheter-Related Bloodstream Infection, VAP: Ventilator-Associated Pneumonia, vHAP: ventilated Hospital-Acquired Pneumonia, HAP: Hospital-Acquired Pneumonia, AST: Antimicrobial Susceptibility Testing, SOFA: Sepsis-related Organ Function Assessment Score, N/A: Not Applicable, SD: Standard Deviation.

|  | **Total (N=83)** | **Group A (N=60)** | **Group B (N=23)** | **p-value** |
| --- | --- | --- | --- | --- |
| Patients with ≥1 adverse event, n (%) | 9 (11) | 2 (3) | 7 (30) | 0.001 |
| Adverse events per patient, median (IQR) | 0 (0-0) | 0 (0-0) | 0 (0-1) | <0.001 |
| Total adverse events, n | 16 | 2 | 14 | NA |
| Renal injury, n (%) | 5 (6) | 0 (0) | 5 (22) | 0.001 |
| Liver injury, n (%) | 3 (4) | 0 (0) | 3 (13) | 0.019 |
| Rash, n(%) | 3 (4) | 1 (2) | 2 (9) | 0.184 |
| Colitis/CDI, n (%) | 2 (2) | 1 (2) | 1 (4) | 0.480 |
| Thrombocytopenia/ hypofibrinogenemia, n (%) | 3 (4) | 0 (0) | 3 (13) | 0.019 |
| Toxicity-associated regimen adjustment/withdrawal, n (%) | 7 (8) | 1 (2) | 6 (26) | <0.001 |
| Colistin | 3 (4) | 0(0) | 3^a^ (13) | 0.004 |
| Ampicillin-sulbactam | 2 (2) | 1^b^ (2) | 1^c^ (4) | 0.476 |
| Meropenem | 0 (0) | 0 (0) | NA | NA |
| Tigecycline | 5 (6) | NA | 5^d^ (22) | NA |

### Table S7. Distribution of safety variables among the two treatment groups. ^a^ All three colistin withdrawals were due to AKI, 6-16 days (range) after colistin initiation. ^b^ ampicillin-sulbactam withdrawal due to gastrointestinal toxicity 26 days after ampicillin-sulbactam initiation, ^c^ ampicillin-sulbactam withdrawal due to rash 4 days after ampicillin-sulbactam initiation, ^d^ 2 cases of liver injury and 1 case each of rash, thrombocytopenia and hypofibrinogenemia, 6-24 days (range) after tigecycline initiation. NA: Not Applicable, CDI: *Clostridioides difficile* infection, AKI: Acute Kidney Injury.

| **Variable** | | **Odds Ratio (95% CI)** | **p-value** |
| --- | --- | --- | --- |
| Site of care (Ward vs ICU) | | 8.235 (1.315-51.578) | 0.024 |
| Infection type | BSI | Reference | 0.110 |
|  | VAP | 0.745 (0.203-2.741) | 0.658 |
|  | vHAP | 7.422 (1.009-54.593) | 0.049 |
|  | HAP | 0.899 (0.115-7.010) | 0.919 |
| SOFA at infection onset | | 1.042 (0.910-1.192) | 0.552 |

### Table S8. Logistic regression model for the prediction of receipt of regimen B. These variables were selected because they varied more between the two groups and could lead to confounding by indication. BSI: Bloodstream Infection, VAP: Ventilator-Associated Pneumonia, vHAP: ventilated Hospital-Acquired Pneumonia, HAP: Hospital-Acquired Pneumonia, SOFA: Sepsis-related Organ Function Assessment.

|  | **Group A (colistin, ampicillin-sulbactam, meropenem)** | **Group B (colistin, ampicillin-sulbactam, tigecycline)** | **p-value** | **SMD** |
| --- | --- | --- | --- | --- |
| n | 82.56 | 76.99 |  |  |
| Age | 67.8 (15.9) | 66.6 (9.5) | 0.703 | 0.089 |
| Female gender | 30.8 (37.3) | 38.2 (49.7) | 0.377 | 0.253 |
| Charlson comorbidity index | 5.0 (3.0) | 4.8 (2.9) | 0.830 | 0.061 |
| McCabe-Jackson classification |  |  | 0.999 | 0.010 |
| Non-fatal disease | 63.4 (76.8) | 58.8 (76.3) |  |  |
| Ultimately fatal disease | 14.0 (16.9) | 13.3 (17.2) |  |  |
| Rapidly fatal disease | 5.2 (6.3) | 4.9 (6.4) |  |  |
| COVID-19 | 18.2 (22.1) | 8.1 (10.5) | 0.284 | 0.319 |
| Respiratory reason for ICU admission | 57.7 (77.4) | 36.2 (54.1) | 0.098 | 0.506 |
| Cardiovascular reason for ICU admission | 37.7 (50.6) | 24.5 (36.6) | 0.377 | 0.285 |
| Chronic kidney disease | 9.6 (11.6) | 9.7 (12.6) | 0.913 | 0.031 |
| Chronic heart failure | 13.2 (16.0) | 15.7 (20.4) | 0.681 | 0.114 |
| Coronary heart disease | 15.6 (18.9) | 23.9 (31.0) | 0.314 | 0.283 |
| Chronic obstructive pulmonary disease | 28.3 (34.2) | 17.2 (22.3) | 0.388 | 0.268 |
| Admission type |  |  | 0.056 | 0.653 |
| Medical | 63.9 (77.4) | 53.3 (69.2) |  |  |
| Elective surgical | 6.1 (7.4) | 0.0 (0.0) |  |  |
| Urgent surgical | 7.6 (9.3) | 21.9 (28.5) |  |  |
| Trauma | 4.9 (5.9) | 1.8 (2.3) |  |  |
| APACHE II score | 19.9 (7.4) | 20.4 (8.8) | 0.841 | 0.059 |
| Time from infection onset to sampling (days) | 0.8 (0.9) | 1.1 (1.2) | 0.357 | 0.277 |
| SOFA score | 7.7 (4.4) | 7.2 (5.1) | 0.685 | 0.113 |
| Pneumonia as infection type | 60.4 (73.1) | 51.8 (67.3) | 0.651 | 0.129 |
| Pneumonia type |  |  | 0.925 | 0.108 |
| VAP | 48.0 (79.5) | 39.8 (76.9) |  |  |
| ventilated HAP | 6.1 (10.1) | 7.0 (13.5) |  |  |
| HAP | 6.3 (10.4) | 5.0 (9.6) |  |  |
| Bacteremic pneumonia | 36.1 (59.8) | 38.4 (74.1) | 0.354 | 0.307 |
| Time from infection onset to AST report (days) | 2.5 (1.2) | 2.2 (1.1) | 0.410 | 0.230 |
| Infection onset maximum temperature (◦C) | 37.9 (0.8) | 38.0 (0.8) | 0.733 | 0.091 |
| Infection onset lower SAP (mmHg) | 101.4 (20.3) | 101.4 (20.3) | 0.999 | <0.001 |
| Infection onset higher heart rate (bpm) | 103.5 (18.2) | 97.8 (18.2) | 0.266 | 0.309 |
| Infection onset higher respiratory rate (bpm) | 23.3 (5.9) | 23.5 (5.2) | 0.898 | 0.035 |
| Infection onset vasopressor administration | 50.3 (60.9) | 36.1 (46.9) | 0.328 | 0.284 |
| IMV at infection onset | 62.4 (75.5) | 43.9 (57.1) | 0.170 | 0.398 |
| CRRT at infection onset | 11.5 (13.9) | 8.2 (10.6) | 0.718 | 0.100 |
| Lactate (mEq/L) | 1.6 (1.1) | 1.8 (2.3) | 0.739 | 0.101 |
| White blood cells (/μL) | 15.2 (8.2) | 13.6 (6.3) | 0.445 | 0.208 |
| Active empiric antimicrobials per patient | 0 (0.0) | 0 (0.0) | N/A | <0.001 |
| Active empiric regimen | 0 (0.0) | 0 (0.0) | N/A | <0.001 |
| Ward as the patient allocation | 10.6 (12.9) | 11.2 (14.6) | 0.839 | 0.050 |
| Sepsis classification |  |  | 0.375 | 0.404 |
| No sepsis | 12.5 (15.2) | 24.3 (31.6) |  |  |
| Sepsis | 36.7 (44.5) | 30.2 (39.2) |  |  |
| Septic shock | 33.3 (40.3) | 22.5 (29.3) |  |  |

### Table S9. Standardized mean differences between treatment groups after the application of inverse probability of treatment weights. Matching was done for patient allocation, infection type and SOFA score at infection onset. Categorical variables are presented as n (%). Numerical variables are presented as mean (SD). COVID-19: Coronavirus Disease 19, ICU: Intensive Care Unit, APACHE: Acute Physiology And Chronic Health Evaluation, SOFA: Sepsis-related Organ Function Assessment, VAP: Ventilator-Associated Pneumonia, HAP: Hospital-Acquired Pneumonia, AST: Antimicrobial Susceptibility Testing, SAP: Systolic Arterial Pressure, bpm: beats/breaths per minute, IMV: Invasive Mechanical Ventilation, CRRT: Continuous Renal Replacement Therapy.

# Supplementary Figures

### Figure S1. Daily disposition and organ support of study population as a total and per treatment group. The left column corresponds to total population, the middle column to group A and the right column to group B. ICU: Intensive Care Unit, IMV: Invasive Mechanical Ventilation, CRRT: Continuous Renal Replacement Therapy.


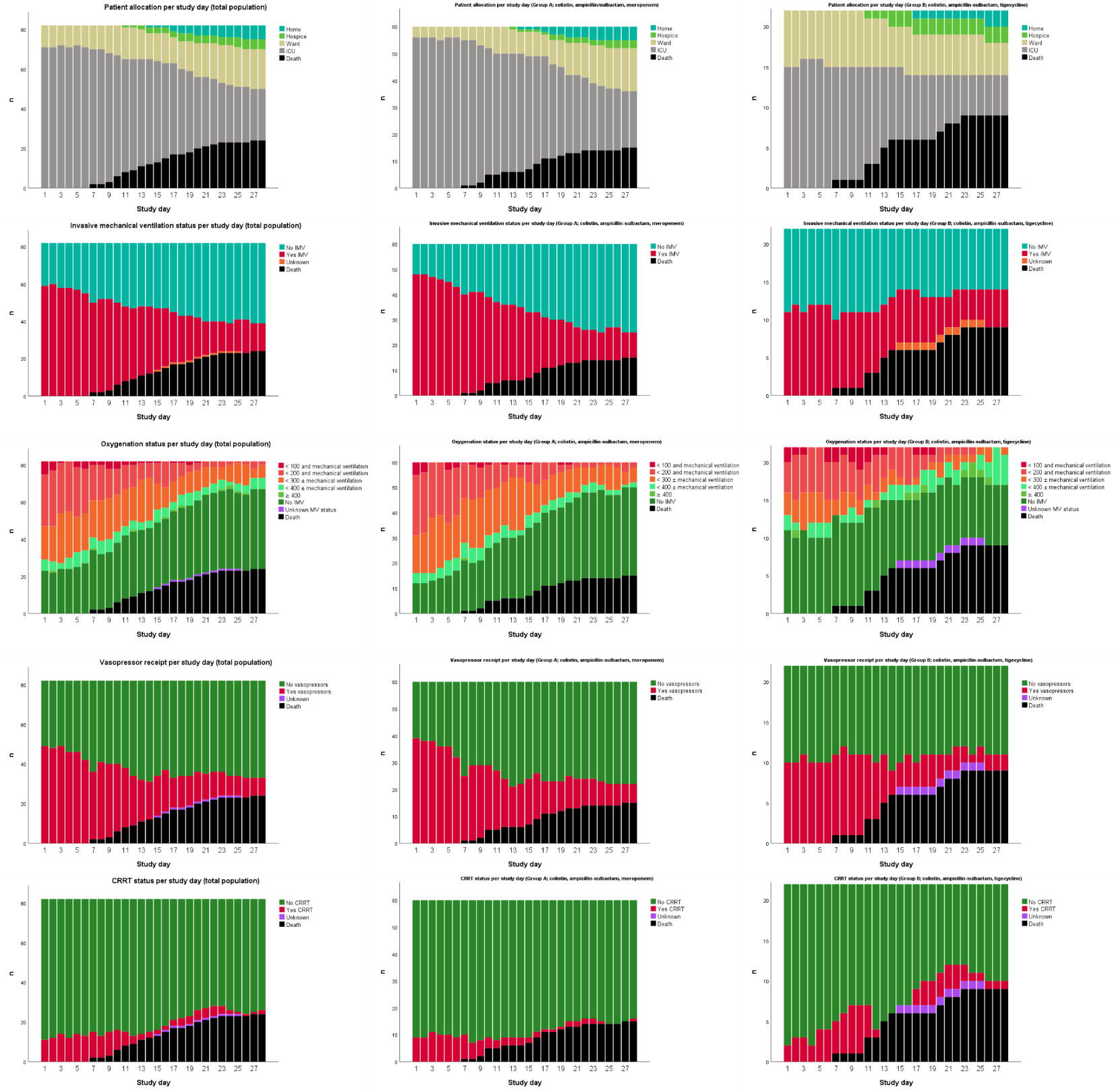


Figure S2. Day 14 survival curves of patients with severe A.baumannii infections.
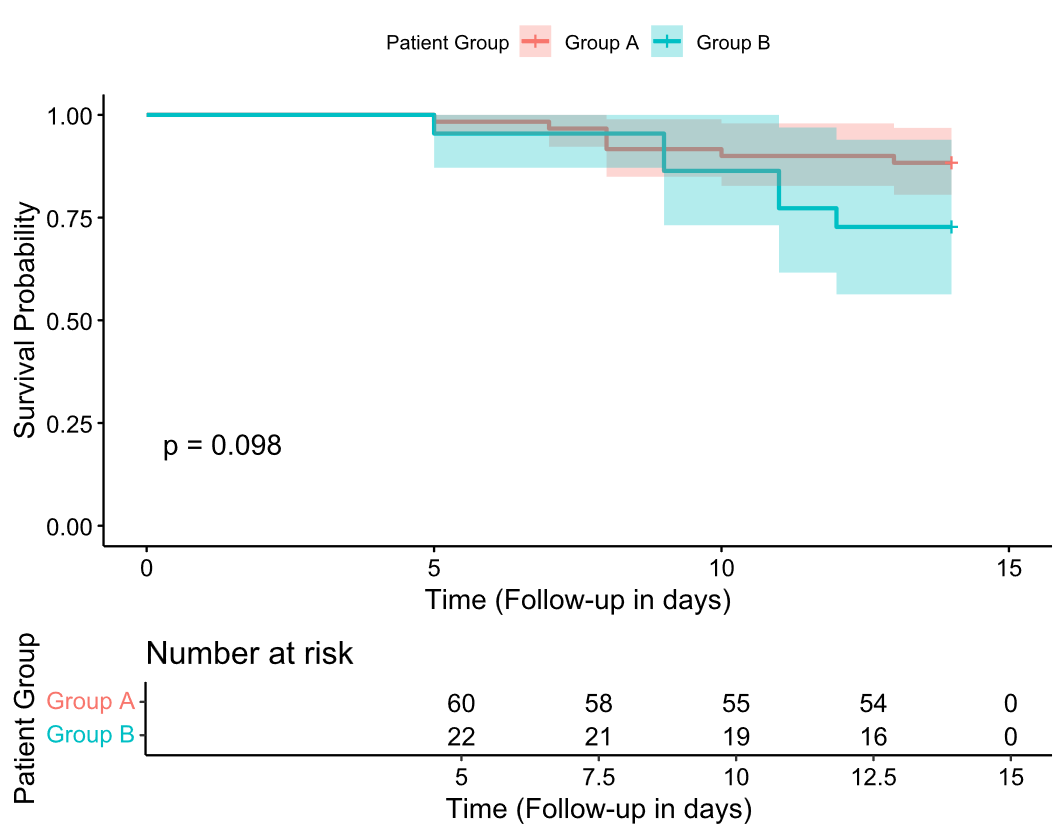


# Supplementary text

Approvals of individual sites are as follows: Chest Diseases Hospital ‘I Sotiria’, Intensive Care Unit 1^st^ Dept of Pulmonology, National & Kapodistrian University of Athens 17797/07-07-2022, Hygeia General Hospital, 1st Department of Internal Medicine - Infectious Diseases 655/15-06-2022, Sismanogleio General Hospital of Attica, Intensive Care Unit 62/15/15-6-2022, Hippokration General Hospital of Thessaloniki, Intensive Care Unit 22-EE-7/28-3-2022, General and Oncologic Hospital of Kifisia ‘Oi Agioi Anargyroi’ 111/16-06-2022, Evangelismos General Hospital 1^st^ Department of Intensive Care Medicine, National & Kapodistrian University of Athens 116/2021, University Hospital of Heraklion, Internal Medicine Department 7/15/15-6-2022, Korgialenio-Mpenakio General Hospital, Intensive Care Unit 760/6-10-2022, Chest Diseases Hospital ‘I Sotiria’, Intensive Care Unit 15189/10-6-2022, University Hospital of Ioannina, Intensive Care Unit 17529/27-6-2024, Hippokration General Hospital, 2^nd^ Department of Internal Medicine, School of Medicine, National & Kapodistrian University of Athens 15696-02/09/2024, Aghios Andreas Hospital, Intensive Care Unit 79/43-2022/8-6-2022
